# Cellulosic hydrolysate toxicity and tolerance mechanisms in *Escherichia coli*

**DOI:** 10.1186/1754-6834-2-26

**Published:** 2009-10-15

**Authors:** Tirzah Y Mills, Nicholas R Sandoval, Ryan T Gill

**Affiliations:** 1 Department of Chemical and Biological Engineering, UCB424/ECCH120, University of Colorado, Boulder, CO 80309, USA

## Abstract

The sustainable production of biofuels will require the efficient utilization of lignocellulosic biomass. A key barrier involves the creation of growth-inhibitory compounds by chemical pretreatment steps, which ultimately reduce the efficiency of fermentative microbial biocatalysts. The primary toxins include organic acids, furan derivatives, and phenolic compounds. Weak acids enter the cell and dissociate, resulting in a drop in intracellular pH as well as various anion-specific effects on metabolism. Furan derivatives, dehydration products of hexose and pentose sugars, have been shown to hinder fermentative enzyme function. Phenolic compounds, formed from lignin, can disrupt membranes and are hypothesized to interfere with the function of intracellular hydrophobic targets. This review covers mechanisms of toxicity and tolerance for these compounds with a specific focus on the important industrial organism *Escherichia coli*. Recent efforts to engineer *E. coli *for improved tolerance to these toxins are also discussed.

## Introduction

Governments around the world are calling for increased production of renewable transportation fuels in light of massive increases in energy consumption [1-5]. The United States has mandated the production of 36 billion gallons of biofuels by 2022, with even greater increases of up to 60 billion gallons by 2030 proposed by the new administration [1,6]. A major challenge is that current production methods based on corn ethanol are limited to 10 to 15 billion gallons per year [7]. Moreover, corn ethanol has recently come under criticism for its potential to increase greenhouse gas emissions when compared to fossil fuels and negative impact on food markets [8-10]. These findings stipulate that new feedstocks and processes capable of producing 20 to 50 billion gallons per year, while not increasing greenhouse gas emissions, must be responsibly developed and commercialized within the next two decades. Biofuels derived from lignocellulosic biomass hold promise for making up a significant fraction of this market.

Lignocellulosic feedstocks, such as switchgrass, poplar, and corn stover, provide greenhouse gas savings of 65 to 100% in comparison to petrol [11]. When land-use changes are considered, cellulosic ethanol still has the ability to reduce overall greenhouse gas emissions depending on the source of biomass and associated land-use change [8]. Feedstocks that do not require a substantial change in land-use include crop and municipal wastes, fall grass harvests, and algae [8]. Other potential feedstocks include waste from pulp and paper mills, construction debris, and animal manures [1]. These feedstocks are of extreme interest because they require no additional land-use conversion [8].

Many processes exist and have been recently reviewed for the pretreatment of lignocellulosic biomass to produce a fermentable hydrolysate [12-16]. The overall goal of pretreatment is to better expose cellulose for downstream hydrolysis, convert hemicellulose to pentoses, and to remove lignin [13].

Harsh conditions used in pretreatment create a variety of toxic compounds that inhibit the fermentation performance. Inhibitors have been categorized previously by Olsson and Hahn-Hägerdal [17]. Specifically, acetic acid is released from acetylxylan decomposition, furan derivatives result from sugar dehydration, and phenolic compounds are derived from lignin. Furan derivatives include 2-furaldehyde (furfural) and 5-hydroxymethylfurfural (HMF), which result from pentose and hexose dehydration, respectively [18,19]. Subsequent degradation of furfural and HMF introduces formic acid and levulinic acid, respectively, into the hydrolysate. Phenolic compounds of interest include acids, alcohols, aldehydes, and ketones [20]. Metallic cation levels have measurable variance depending on the pretreatment method, but levels are low enough to not significantly affect fermentation [21].

Although many fermentative microorganisms exist, *Escherichia coli*, *Saccharomyces cerevisiae*, and *Zymomonas mobilis *are the most promising industrial biocatalysts for biofuels production [22]. Each microorganism has limitations in native substrate utilization, production capacity, or tolerance. Unlike *S. cerevisiae *or *Z. mobilis*, *E. coli *natively ferments both hexose and pentose sugars. Ethanologenic *E. coli *also has higher tolerance to lignocellulosic inhibitors than its fermentative counterparts [23-25]. In 2007, Jarboe *et al*. [26] compared ethanol production between these three microorganisms, determining that *E. coli *is comparable with or surpasses other reported production levels, despite its low membrane tolerance to ethanol. These qualities along with advanced knowledge about the *E. coli *genome and regulation make this bacterium a prime candidate for further development.

**Figure 1:**
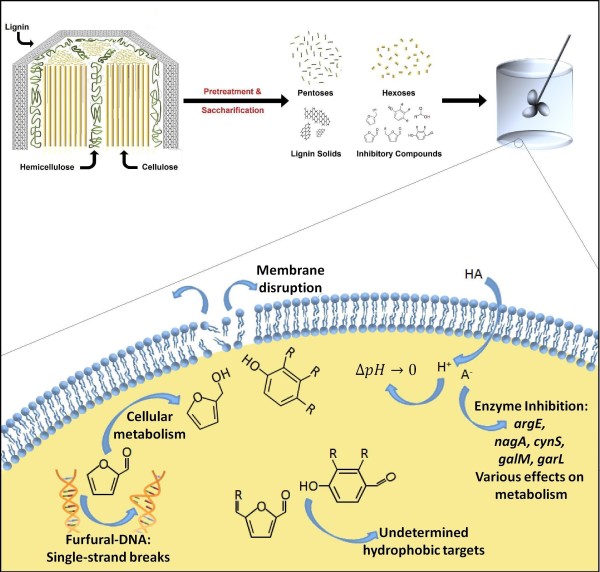
**Hydrolysate inhibitors**. Lignocellulosic biomass is processed into component sugars, lignin solids, and inhibitory compounds. These inhibitors can affect microbial growth in various ways, including DNA mutation, membrane disruption, intracellular pH drop, and other cellular targets.

As depicted in Figure 1, generally accepted categories of antimicrobial activity for inhibitors in lignocellulosic hydrolysate include: compromising the cell membrane; inhibiting essential enzymes; or negative interaction with DNA or RNA [27-32]. These compounds often act by inhibiting multiple targets. Although efforts are underway to limit the amount and types of inhibitors created during pretreatment, at the present time, economically viable processes still fall short. Regardless of pretreatment optimization, inhibitors such as acetic acid, released directly from hemicellulose decomposition, will remain in the hydrolysate. Thus, the need to engineer more tolerant fermentative microorganisms exists. In this work, known modes of toxicity and tolerance pertaining to *E. coli *and lignocellulosic inhibitors will be reviewed, in addition to new technologies that are aimed at engineering the bacterium for fermentation of lignocellulosic biomass.

## Organic acids

Organic acids derived from lignocellulosic biomass pretreatment and subsequent saccharification inhibit the growth and metabolism of *E. coli*. This, in turn, reduces the yield, titer, and productivity of biofuel fermentation. Various organic acids are created in pretreatment steps: acetic acid is derived from the hydrolysis of acetylxylan, a main component of hemicellulose; others (formic, levulinic, and so on) are result from degraded sugars [33].

Acetic acid is usually found at the highest concentration in the hydrolysate [34-40]. Levels of acetate depend on the type of cellulosic biomass and the pretreatment method. Concentrations typically range from 1 to >10 g/L in the hydrolysate. Formic acid, while more toxic to *E. coli *than acetic acid, is typically present at concentrations much less than that of acetic acid (commonly a tenth of acetic acid concentrations) [23,35,36]. Other toxic weak acids, whose hydrolysate concentrations are rarely reported, are present at an even lower concentration than formic acid [35,37,38,41].

### Modes of toxicity

Weak organic acids have been shown to primarily inhibit the production of cell mass, but not the fermentation itself [23]. Acetate is the most studied organic acid inhibitor in *E. coli*. Acetate is a natural fermentation product that is known to accumulate due to 'overflow metabolism' and inhibit cell growth. Acetate concentrations as low as 0.5 g/L have been shown to inhibit cell growth by 50% in minimal media [42,43]. However, in *E. coli *KO11, concentrations of acetate up to 12 g/L did not significantly affect ethanol yield, although ethanol titer decreased with high levels of acetate [44]. Attempts have been made to mathematically describe the relationship between growth rate and acetate concentration, with varying results. Koh *et al*. [45] proposed the following equation for specific growth (μ) in a batch reactor:



The value of the constant, *k*, ranged from 0.125 L/g to 0.366 L/g depending on the strain and media [45]. Luli and Strohl [46] reported an exponential decay model of inhibition:



The value of the constant was calculated as 0.06 L/g of acetate. In both shake flasks and a fermentor, Nakano *et al*. [47] report a linear inhibition trend. Specific growth rates in shake flasks were four times as low for any given concentration of acetate compared to the fermentor. This difference in toxicity was attributed to the controlled dissolved oxygen in the fermentor. The IC_50_, the concentration of acetate that inhibits growth by 50%, ranges from 2.75 to 8 g/L depending on the strain and media [23,45,46].

Weak acids in the undissociated form can permeate the cell membrane, and, once inside, dissociate to release the anion and the proton. These 'uncoupling agents' disrupt the transmembrane pH potential since, effectively, a proton is allowed across the membrane without the creation of ATP [48]. This dissociation of the weak acid inside the cytoplasm is due to the fact the intracellular pH, pH_*i*_, is naturally at a pH of approximately 7.8, which is much higher than the weak acid's pK_*a *_[42]. As these acids dissociate inside the cell, the pH_*i *_decreases, which can inhibit growth [42]. External pH has a large affect on the toxicity of the weak acids. *E. coli *KO11 in LB media with 5.0 g/L acetate reached an ethanol titer twice as fast at an initial pH of 7.0 compared to initial pH of 6.0, and thrice as fast compared to an initial pH of 5.5 [44]. When *E. coli *LY01 was subjected, at a starting pH of 6.0, to acetic, formic, or levulinic acid at the IC_50 _obtained at a neutral pH, the growth rate decreased to 0%, 35%, and 10%, respectively, that of control growth [23]. Formic acid may be more toxic due to the fact it has an extraordinarily high permeability through the membrane [49]. This external pH effect is due, in part, to the fact that the acid exists in its undissociated form at higher concentrations, allowing for higher permeation of the cell membrane.

The anion also has an inhibitory effect. The anion accumulates inside the cell, which can affect the cell turgor pressure [42]. Inhibition has been shown to be anion specific [23,42,43]. When *E. coli *inhibition from acetate was compared to benzoate, the same growth rate was observed for differing pH_*i *_(7.26 for benzoate and 7.48 for acetate) [42]. Zaldivar and Ingram [23] reported that the toxicity of weak acids depended highly on the hydrophobicity of the acid.

The modes of toxicity of weak acids are not easily elucidated. Formic and propionic acid have been shown to inhibit the synthesis of macromolecules, as the cells stop growing after addition of the acids [50]. More so than other macromolecules, DNA synthesis was slowed [50]. DNA repair-deficient strains were shown to be more sensitive to weak acids when tested in stationary phase [51]. However, repair deficient strains were not overly sensitive to organic acids in growth phase [52]. This, plus the lack of an observed SOS response, suggests that the DNA was not damaged by these acids [52]. The hypothesis of membrane disruption has also been investigated. Leakage of cell contents in the presence of weak organic acids was small when compared to the leakage associated with a membrane disrupting antibiotic (polymyxin B) or even ethanol, and thus is not likely to be the primary cause of weak acid inhibition [23,53]. Weak acids have been shown to reduce the intracellular pools of some amino acids. Glutamate and aspartate, precursors to many different amino acids, were shown to be at a significantly lower concentration in the cytoplasm when *E. coli *was grown in the presence of weak acid [42]. Glutamate has been shown to be important during growth as a protective osmolyte [54,55]. Lysine, arginine, glutamine, and methionine were also found at lower concentrations when incubated with weak acid [42,43]. The addition of methionine to the incubation mixture has been shown to alleviate much of the toxicity associated with acetate [43].

### Modes of tolerance

*E. coli *acid resistance mechanisms are thought to increase *E. coli *survival when passing through the low pH environment in the stomach. It has long been known that cells can sense and regulate intracellular pH [56]. Also, it has been shown that treatment of bacteria to moderately low levels of pH (5.0) before exposure to very low pH (3.0 to 3.5) increases the tolerance more than 50-fold [57].

*E. coli *naturally has several known mechanisms to combat acid stress. One mechanism for acid tolerance requires the presence of an amino acid decarboxylase coupled with an antiporter that exports the decarboxylated product and imports the amino acid used [58-60]. It is widely thought that the tolerance is due to the fact that the decarboxylation and antiporter reactions consume and export one intracellular proton across the cell membrane. This raises the pH_*i *_of the cell, which is beneficial for survival and growth [58-62]. The transmembrane potential is also affected by these acid resistance mechanisms. *E. coli*, which normally has a negative transmembrane potential, had a positive potential during acid stress when either the arginine- or glutamine-dependent systems were activated. This mimics what is seen in acidophiles [61]. These mechanisms of tolerance have also been reviewed and depicted by Warnecke *et al*. [63].

All acid resistance mechanisms, however, are not equally effective. The glutamate-dependent acid resistance mechanism is the most studied and the most robust, the arginine-dependent mechanism provides a moderate level of resistance, and the lysine-dependent mechanism confers a minimal level of tolerance [59-62,64,65]. The levels of tolerance are highly dependent on the strain, treatment before shock, the media used, growth phase, and the strength and length of acid stress [58-62,64,65]. The differences in efficacy between the mechanisms may lie in the optimal pH for the amino acid decarboxylase. The optimum pHs for the glutamate, arginine, and lysine decarboxylases are 4, 5, and 5.7, respectively [61]. The lower the optimal pH of the enzyme, the more efficient it is during times of acid stress.

These acid resistance mechanisms have complicated regulation. Low pH can induce heat and oxidative shock regulons, genes coding for membrane-proteins, and acid consumption [66]. It is known that the *rpoS *regulon is induced by exposure to weak acids [64,67,68]. Once induced, the *rpoS *response leads to higher survival rates at low pH, oxidative stress, and heat stress [68]. However, the *rpoS *response alone is not sufficient for acid tolerance. Cultures exposed to NaCl, which also induced the *rpoS *response, failed to increase acid survival [68]. *RpoS *has also been implicated in glutamine-dependent acid resistance [62]. This system has been shown to have at least two sigma factors (σ^S ^and σ^70^) and at least five regulatory proteins (encoded by *crp*, *ydeO*, *gadE*, *gadX*, and *gadW*) involved in the expression of the decarboxylases (*gadA *and *B*) and the antiporter (*gadC*) [69-71].

Other modes of tolerance to weak acids are also known. DNA stabilization via Dps protein interactions has been shown to be beneficial at low pH [72]. Acetate treatment was shown to increase expression of many other genes; these genes are mostly involved in general metabolism of the cell as well as in outer membrane protein production [68]. In a genomic library selection with 3-hydroxyproionic acid, genes coding for inner membrane proteins and certain genes involved in cell metabolism were found to be most enriched [73,74].

## Furan derivatives

Furan derivatives are a result of sugar dehydration during pretreatment. Furfural and HMF are the primary derivatives appearing in lignocellulosic hydrolysate. Concentrations typically range between 0 and 5 g/L for each compound [20,21,75,76]. As previously mentioned, levulinic and formic acid are also formed via degradation of these aldehydes [77]. While dilute acid hydrolysis is a common method for pretreatment, acidic conditions are known to cause dehydration of a small fraction of the sugar monomers. Hemicellulose is the second most abundant renewable polysaccharide, averaging 25 to 35% of viable lignocellulosic biomass composition [78]. Therefore, processes that avoid degradation of the C_5 _and C_6 _monomers are vital. While new methods are being developed to reduce the amount of furfural and HMF formed during pretreatment [79-81], industrial-scale technology and knowledge about process kinetics currently favors more traditional processes like dilute sulfuric acid treatment [34,82,83]. Therefore, it is important to improve understanding of the genetic and metabolic mechanisms underlying tolerance to furan derivatives.

Aldehydes in general are known to have detrimental effects in microorganisms. For example, Haselkorn and Doty [84] showed that formaldehyde denatures and interacts with polynucleotides. Formaldehyde is also known to cause protein-protein cross-linking [85]. *In vitro *experiments with crude cell extracts identified a glutathione-dependent formaldehyde dehydrogenase that is responsible for conferring aldehyde tolerance [86]. Two previously uncharacterized proteins, FrmB and YeiG, have also been identified for their role in conferring folrmaldehyde tolerance via a glutathione-dependent formaldehyde hydrolysis pathway [87]. Besides enzymatic detoxification, outer membrane protein composition has also been indicated as conferring increased tolerance to formaldehyde, acetylaldehyde, and glutaraldehyde [88]. Furthermore, methylgloxal, a dicarbonyl compound, has been shown to inhibit *E. coli *growth and protein synthesis at concentrations of 0.07 g/L [89,90]. We will focus here on the two primary aldehyde compounds found in the hydrolysate, furfural and HMF.

### Modes of toxicity

Furfural has been identified as a key inhibitor in lignocellulosic hydrolysate because it is toxic by itself and also acts synergistically with other inhibitors [24]. Hydrophobicity is a marker of an organic compound's toxicity. Highly hydrophobic compounds have been shown to compromise membrane integrity [29]. Interestingly, perceptible membrane damage in *E. coli *resulting from furfural exposure has not been observed, despite a known log(P_octanol/water_) value of 0.41 [24]. Intracellular sites are more likely to be the primary inhibition targets of furfural and HMF. In contrast, both 2-furoic acid and furfuryl alcohol have been shown to cause significant membrane leakage [23,25]. Furfuryl alcohol also exhibits synergism when in binary combinations with other inhibitors, while 2-furoic acid results in additive toxicity [23,24].

Ethanol production is inhibited in *E. coli *LYO1 by furfural, suggesting a direct effect on glycotic and/or fermentative enzymes [24]. Glycotic dehydrogenases like alcohol dehydrogenase (ADH) have been indicated as a potential site of inhibition via NAD(P)H-dependent aldehyde reduction into the corresponding alcohol [27]. A study performed *in vitro *has confirmed that acetaldehyde to ethanol conversion was inhibited by both furfural and HMF [91]. Subsequent *in vitro *enzymatic assays in this study demonstrated that furfural was a substrate for ADH (EC 1.1.1.1), albeit at a five-fold increase in *K*_*m *_and five-fold decrease in *V*_*max*_. In the same study, furfural inhibition on aldehyde dehydrogenase (EC 1.2.1.5) and the pyruvate dehydrogenase complex were investigated and determined to be more significant than ADH, as indicated by more than 80% activity reduction in the presence of 0.12 g/L furfural, whereas ADH activity was only inhibited by 60%. These findings suggest that furfural may detrimentally affect multiple glycotic enzymes essential to central metabolism.

Furfural and HMF have shown cytotoxic characteristics towards both bacteria and yeast [24,92-94]. Furfural is a known dietary mutagen and has been under investigation for direct effects on DNA in the past. A series of studies by Hadi and coworkers confirmed that furfural-DNA interactions occur. Furfural-treated double-stranded DNA led to single-strand breaks after undergoing *in vitro *incubation with furfural, primarily at sequence sites with three or more adenine or thymine bases [28,32]. Later, plasmids treated with furfural were observed to cause either an increase (high furfural concentrations) or decrease (low furfural concentrations) in plasmid size via insertions, duplications, or deletions [30].

### Modes of tolerance

Although furfural damages DNA, cells with necessary DNA repair mechanisms still maintain viability. Despite the mutagenic interaction of furfural with DNA as previously stated, *in vivo *experimentation suggests the importance of the *polA*-mediated DNA repair pathway for tolerating scissions caused by furfural [31]. Cells have been observed to repair damaged DNA, reducing the frequency of furfural-induced mutagenic events to that of random mutation found in untreated cultures [95].

Recombinant *E. coli *has been shown to metabolize furfural into furfuryl alcohol under aerobic conditions [96]. The bioconversion is thought to occur via a NADPH-dependent furfural reductase, which is the first of its kind to be reported in the class of alcohol-aldehyde oxidoreductases [97]. In the same study, the furfural reductase showed an increased rate of NADPH oxidation when acting on benzaldehyde compared to furfural, suggesting that it can utilize a variety of aldehydes as substrates.

Conversely, a recent long-course adaptation experiment with ethanologenic *E. coli *found that furfural tolerance is conferred by silencing certain NADPH-dependent oxioreductases [98]. Genes of special interest in this work were *yqhD *and *dkgA*, both of which encode gene products with low *K*_*m*_s for NADPH, allowing for biosynthetic reaction competition. Miller *et al*. [98] propose that competition exists between furfural reduction and biosynthesis by observing that cells initially undergo a lag phase, consistent with decreased biosynthesis, as the NADPH pool is devoted to furfural reduction. The mutant with silenced *yqhD *and *dkgA *genes was able to concurrently reduce furfural and grow, providing further support for the proposed claim. YqhD is also reported to play an important role in protecting *E. coli *from aldehydes derived from lipid-peroxidation via a glutathione-independent, NADPH-dependent reduction mechanism [99]. Interesting to note is that the mutant obtained from this study also overexpressed eight oxioreductases that can use NADPH as an electron donor. For example, the product of one such gene, *yajO*, is highly specific for utilizing 2-carboxybenzaldehyde as a substrate in comparison to a variety of other aldehydes [100]. Further studies should be performed on these isolated oxioreducatases for a variety of substrates to explore the interplay between them and the detrimental effects of cellular utilization of NADPH for aldehyde reduction because NADH- and NADPH-dependent reduction of furan derivatives has proved paramount for hydrolysate inhibitor tolerance in *S. cerevisiae *and *Pichia stipitis *[101-105].

*E. coli *K12 mutants are also capable of converting furfural to 2-furoic acid, a weak acid that can form at low levels during pretreatment [106,107]. This acid inhibits growth at concentrations as low as 0.5 g/L [23,24]. Interestingly, these *E. coli *K12 mutants were shown to metabolize 2-furoic acid and furfuryl alcohol as sole carbon sources [108]. Isolated mutants from this study revealed beneficial mutations in *atoC *and *fadR*, genes related to transcriptional activation and regulation of fatty acid metabolism [109,110]. The mechanism relating fatty acid metabolism with furfural metabolism has not yet been determined.

## Phenolic compounds

Hydrolysates can contain up to 30% lignin content for a variety of feedstocks [83,92]. Major phenolic compounds have carboxyl, formyl, or hydroxyl group functionalities and arise from degradation of lignin during pretreatment. Ketones can also be released during pretreatment, but are not generally considered as primary inhibitors because they occur at low concentrations (<0.05 g/L) and are also partially or completely removed with various detoxification treatments [20]. Most of the lignin and its derivatives are insoluble; after dilute acid pretreatment of yellow poplar, no more than 15% of the total lignin feedstock content was converted to a soluble species [33,92]. Concentrations of aromatic monomers after dilute acid washes have been measured at between 0 and 3 g/L and include acids, alcohols, and aldehydes [20,111,112]. Due to the number of lignin-derived compounds needing to be analyzed, sequential studies with *E. coli *have been limited. As such, only the most commonly studied compounds are reviewed in this work.

### Modes of toxicity

A series of studies comparing aldehydes, acids, and alcohols appearing in hydrolysate were performed with the ethanologenic *E. coli *LYO1 [23-25]. In general, the degree of toxicity correlated with the compound's octanol/water partition coefficient, log(P_octanol/water_), which is a measure of hydrophobicity. In all studies the phenolics were more toxic than aliphatics or furans with the same functional group. This observation that hydrophobicity was related to membrane damage was only true for the alcohols tested, with the exception of hydroquinone. Aromatic acids caused partial membrane leakage while the aromatic aldehydes caused no significant membrane damage. A synergistic binary combination was observed for guaiacol and methylcatechol, but a less than additive combination was observed for vanillyl alcohol and all lignin-derived alcohols tested (catechol, coniferyl, guaiacol, hydroquinone, and methylcatechol). Vanillin, a phenolic aldehyde, was found to be bacteriostatic and membrane active, thus causing partial disruption of K^+ ^gradients in *E. coli *MC1022 [113]. This finding is similar to the effect of methylglyoxal on *E. coli *[114]. Membrane destabilization was experienced by 29% of the population after treatment with vanillin for 1 hour at over three times the minimum inhibitory concentration, but restored to 13% when grown overnight [113]. In addition, this study showed that ATP production continues without significant interruption. In previous reports, membrane damage was found to not contribute significantly to toxicity [24]. From these data, hypotheses have been developed stating that other cellular hydrophobic components may be the primary target for inhibition [24,113].

### Modes of tolerance

From the studies conducted on *E. coli *LYO1, only tolerance to aldehydes benefited from increased inoculum size, suggesting metabolism of the compounds [23-25]. Similar to findings on furan derivatives, microbial metabolism of phenolic aldehydes is supported by previous findings in recombinant *E. coli*, the closely related enteric bacterium *Klebsiella pneumonia*, and *S. cerevisiae *[97,115-117]. Furthermore, recombinant *E. coli *are capable of converting aromatic aldehydes to their corresponding acids [118]. Non-lignin derived aromatic acids have also been shown to be metabolized as sole carbon sources, similar to observations of furfural and HMF metabolism [108]. Conversion of an aldehyde to carboxylic acid or alcohol is often beneficial for *E. coli *due to the reduced toxicity of the functional group [23-25]. To date, tolerance to phenolic compounds has not been adequately studied for Gram-negative prokaryotes. A recent study on *S. cerevisiae *identified genes required for vanillin tolerance, but these genes are categorized for chromatin remodeling and vesicle transport functionalities, which does not readily lend itself to application for *E. coli *[119].

## Engineering tolerance

Engineering tolerance to hydrolysate byproducts is an attractive method for improving lignocellulosic biomass based biofuel production in *E. coli*. Several methodologies have been used for this purpose. The conventional approach is to perform long-course adaptation studies. This method has been used to generate the ethanologenic *E. coli *LY01 strain. Over a 3-month period, *E. coli *KO11 was grown recursively in ethanol-containing media and plated on chloramphenicol-containing solid media (on which large colonies indicated good ethanol production) [120]. The LY01 strain showed 50% relative growth rate (μ) at 30 g/L ethanol where the parent KO11 showed 50% relative growth rate (μ) in 20 g/L [24]. The resultant *E. coli *LY01 strain was not only more tolerant to ethanol than KO11, but showed a decreased sensitivity to toxic aldehydes as well [24]. Gonzalez *et al*. [121] showed expression levels of genes involved in protective osmolytes, antibiotic resistance proteins, and cell envelope components were significantly different in LY01 and KO11. Using chemical mutagens can, over a short period of time, achieve similar results as long-course adaptation. Randomly mutating *E. coli *using nitrosoguanidine mutagenesis has been used to increase the complete inhibition concentration of vanillin from 3 to 4 g/L [122].

Genomic library selection is a powerful tool that can discover genes or operons that, with increased copy number, confer a desired phenotype. The advent of DNA microarrays has made it easier to identify these beneficial genes. SCALEs (Scalar Analysis of Library Enrichments), and its predecessor PGTM (Parallel Gene Trait Mapping), have used *E. coli *genomic library selection and microarrays to engineer tolerance to Pine-Sol antibiotic, antimetabolites, 3-hydroxypropionic acid, and naphthol [73,123-126]. Genomic selections employing libraries of heterologous genes have also been used to engineer tolerance. A genomic library of *Sphingomonas *sp. 14DN61 was used in *E. coli *to find the PhnN enzyme, which converts aromatic aldehydes, such as vanillin, to their milder corresponding carboxylic acid [118]. Other methods of creating tolerant strains include engineering sigma factors, which alter the transcription of the cell. Global transcription machinery engineering utilizes random mutagenesis of sigma factor genes to create libraries of mutated sigma factors. These mutants are then selected for improved tolerance. As a proof of concept, a 40% increase in growth rate at 40 g/L ethanol tolerance was reported [127]. Also, mutants found after global transcription machinery engineering selection using high levels of acetate (30 g/L) increased growth rate (μ) by a factor of five [128].

Rational designing of *E. coli *to better cope with toxins in hydrolysate has yielded mixed results. After determining methionine biosynthesis was being inhibited in the presence of acetate, Roe *et al*. [43] overexpressed the *metE *gene, which converts homocysteine to methionine, and the *glyA *gene, which is necessary for 5N-methyltetrahydrofolate regeneration (a part of methionine synthesis). However, no decrease in acetate sensitivity was found with either clone. Heterologous cloning of potentially beneficial genes has also been attempted. Aldehyde oxidoreductase from a *Nocardia *species reduces aromatic carboxylic acids to the corresponding aldehydes, which are then natively converted to the milder corresponding alcohol. This gene was cloned in to *E. coli*, but a 50-fold lower specific activity was seen [129]. When incubated with a cofactor and the *Nocardia *sp. post-translation enzyme, heterologous expression gave a specific activity 20-fold higher than before [129]. In another effort, *Pseudomonas putida *benzaldehyde dehydrogenase was cloned into *E. coli*. Coupled with a NahR reporter system, catalytically active enzyme was selected for using a tet-based host [130]. The fungus *Coniochaeta ligniaria *was found by selection of various microorganisms sampled from soil in media containing furfural and HMF. It was later shown to degrade both furfural and HMF [79]. The genes responsible for such degradation may be attractive metabolic engineering targets. In a novel fermentation strategy, Eiteman *et al*. [131] propose using *E. coli *strains designed to be able to use only one substrate as a carbon source. In a two-part fermentation, a strain designed to consume only acetate acts first, then, the detoxified hydrolysate would undergo simultaneous fermentation by a glucose-consuming strain and a xylose-consuming strain [131,132].

## Conclusion

Biofuels production must find cost-effective and sustainable feedstocks. The commercial potential of biofuels largely depends on the abundance and cost of the feedstock. From 2000 to 2007, global biofuel production tripled, but is still only 3% of the global transportation energy [133]. As this number grows, commercial processes will necessarily rely more heavily upon lignocellulosic biomass. Much work is still required to improve the efficiency of fermentations of biomass hydrolysate to levels cost competitive with fermentation of pure sugar streams. Emphasis should be placed upon not only further reducing the cost of the enzymatic hydrolysis step but also upon better understanding of hydrolysate toxicity mechanisms and methods for engineering tolerance. More specifically, elucidating the modes of action of specific compounds present in hydrolysate will prove critical since the levels of inhibition of various aldehydes and weak acids can vary greatly. It is for this reason that new technologies must emerge in order to more rapidly decipher toxicity and tolerance phenotypes. Once such understanding is generated, processes involving fermentation of lignocelluosic hydrolysates that meet and surpass the productivity of sugar-based bioprocesses will be enabled.

## Abbreviations

ADH: alcohol dehydrogenase; HMF: 5-hydroxymethylfurfural.

## Competing interests

The authors declare that they have no competing interests.

## Authors' contributions

TYM authored the Introduction, Furan derivatives, and Phenolic compounds portions of this manuscript. NRS authored the Organic acids and Engineering tolerance sections and the Conclusions. RTG provided critical review, structural advice, as well as writing and editing.
